# The role of FDG PET/CT in patients treated with neoadjuvant chemotherapy for localized bone sarcomas

**DOI:** 10.1007/s00259-016-3509-z

**Published:** 2016-09-20

**Authors:** Emanuela Palmerini, Marco Colangeli, Cristina Nanni, Stefano Fanti, Emanuela Marchesi, Anna Paioli, Piero Picci, Silvia Cambioli, Davide Donati, Luca Cevolani, Massimiliano De Paolis, Marco Gambarotti, Stefano Ferrari

**Affiliations:** 1 Chemotherapy, Istituto Ortopedico Rizzoli, Via Pupilli 1, 40136 Bologna, Italy; 2 Orthopaedic Surgery, Istituto Ortopedico Rizzoli, Via Pupilli 1, 40136 Bologna, Italy; 3 Nuclear Medicine, Sant’ Orsola Hospital, Bologna, Italy; 4 Research Laboratory, Istituto Ortopedico Rizzoli, Via Pupilli 1, 40136 Bologna, Italy; 5 Surgical Pathology, Istituto Ortopedico Rizzoli, Via Pupilli 1, 40136 Bologna, Italy; 6 Radiology, Musculoskeletal Oncology Department, Istituto Ortopedico Rizzoli, Via Pupilli 1, 40136 Bologna, Italy

**Keywords:** Ewing sarcoma, Osteosarcoma, PET-CT, Prognosis, Neo-adjuvant chemotherapy, SUV1

## Abstract

**Purpose:**

The histological response to neoadjuvant chemotherapy is an important prognostic factor in patients with osteosarcoma (OS) and Ewing sarcoma (EWS). The aim of this study was to assess baseline primary tumour FDG uptake on PET/CT, and serum values of alkaline phosphatase (ALP) and lactate dehydrogenase (LDH), to establish whether these factors are correlated with tumour necrosis and prognosis.

**Methods:**

Patients treated between 2009 and 2014 for localized EWS and OS, who underwent FDG PET/CT as part of their staging work-up, were included. The relationships between primary tumour SUVmax at baseline (SUV1), SUVmax after induction chemotherapy (SUV2), metabolic response calculated as [(SUV1 − SUV2)/SUV1)] × 100, LDH and ALP and tumour response/survival were analysed. A good response (GR) was defined as tumour necrosis >90 % in patients with OS, and grade II-III Picci necrosis (persitence of microscopic foci only or no viable tumor) in patients with Ewing sarcoma.

**Results:**

The study included 77 patients, 45 with EWS and 32 with OS. A good histological response was achieved in 53 % of EWS patients, and 41 % of OS patients. The 3-year event-free survival (EFS) was 57 % in EWS patients and 48 % OS patients. The median SUV1 was 5.6 (range 0 – 17) in EWS patients and 7.9 (range 0 – 24) in OS patients (*p* = 0.006). In EWS patients the GR rate was 30 % in those with a high SUV1 (≥6) and 72 % in those with a lower SUV1 (*p* = 0.0004), and in OS patients the GR rate was 29 % in those with SUV1 ≥6 and 64 % in those with a lower SUV1 (*p* = 0.05). In the univariate analysis the 3-year EFS was significantly better in patients with a low ALP level (59 %) than in those with a high ALP level (22 %, *p* = 0.02) and in patients with a low LDH level (62 %) than in those with a high LDH level (37 %, *p* = 0.004). In EWS patients the 3-year EFS was 37 % in those with a high SUV1 and 75 % in those with a low SUV1 (*p* = 0.004), and in OS patients the 3-year EFS was 32 % in those with a high SUV1 and 66 % in those with a low SUV1 (*p* = 0.1). Histology, age and gender were not associated with survival. In the multivariate analysis, SUV1 was the only independent pretreatment prognostic factor to retain statistical significance (*p* = 0.017). SUV2 was assessed in 25 EWS patients: the median SUV2 was 1.9 (range 1 – 8). The GR rate was 20 % in patients with a high SUV2, and 67 % in those with a low SUV2 (*p* = 0.02). A good metabolic response (SUV reduction of ≥55 %) was associated with a 3-year EFS of 80 % and a poor metabolic response with a 3-year EFS of 20 % (*p* = 0.05). In the OS patients the median SUV2 was 2.7 (range 0 – 4.5). Neither SUV2 nor the metabolic response was associated with outcome in OS patients.

**Conclusion:**

FDG PET/CT is a useful and noninvasive tool for identifying patients who are more likely to be resistant to chemotherapy. If this finding is confirmed in a larger series, SUV1, SUV2 and metabolic response could be proposed as factors for stratifying EWS patients to identify those with high-grade localized bone EWS who would benefit from risk-adapted induction chemotherapy.

## Introduction

Osteosarcoma (OS) and Ewing sarcoma (EWS) are the most frequent primitive bone tumours, with an incidence ranging from 0.2 to 0.3/100,000/year [1, 2]. The combination of multiagent chemotherapy, surgery and also radiotherapy in patients with EWS have dramatically improved the prognosis, with disease-free survival rates at 5 years of about 65 – 70 % [1]. Tumour necrosis induced by neoadjuvant chemotherapy is one of the most powerful prognostic indicators of survival in patients with localized disease [3, 4]. Tumour response to neoadjuvant chemotherapy has important implications in subsequent patient management and some clinical trials have indicated that postoperative treatment should be based on histological response [5, 6]. However, pathological assessment of tumour response is only possible after resection. Therefore, an accurate and noninvasive predictive marker of response is important in designing an individualized treatment strategy in patients with localized bone sarcoma. This is particularly relevant in patients with nonextremity EWS who are undergoing radiotherapy as definitive treatment, and therefore lack histological response data [7, 8].

^18^F-FDG PET/CT is now widely used in the initial diagnosis, staging and detection of recurrence in many kinds of cancer [9–14]. The role of ^18^F-FDG PET/CT in predicting response to chemotherapy in bone sarcomas [15–22] and soft-tissue sarcomas [23–25] has been assessed in many studies with contradictory results [19, 26]. However, histological heterogeneity [23, 24], limited numbers of patients included [16, 19], and especially the lack of uniform treatment [15] make the interpretation of results difficult.

The aim of the present study was to assess the prognostic role of ^18^F-FDG uptake and its correlation with histological response to chemotherapy, in a single-institutional series of patients with EWS and OS of bone prospectively enrolled in a clinical trial (for EWS, EudraCT no. 2008-008361-35; for OS, EudraCT no. 2011-001659-36).

## Materials and methods

All patients treated between April 2009 and February 2014 for localized EWS and OS enrolled in the ISG-AIEOP-EW1 and ISG/OS2 protocols, respectively and who had undergone FDG PET/CT as part of staging work-up were included.

### EWS and OS protocol design

**Fig. 1:**
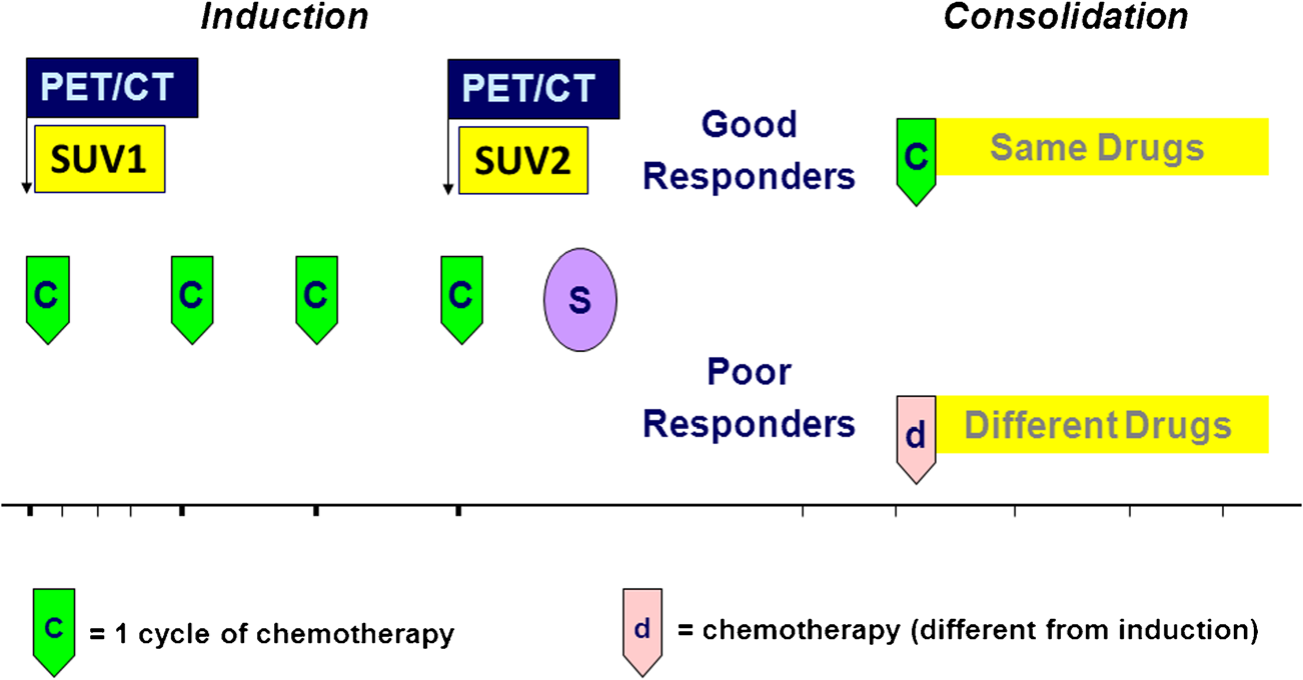
Chemotherapy treatment schedule for the treatment of patients with localized bone tumours

EWS patients enrolled in the ISG-AEIOP-EW1 protocol (EudraCT no. 2008-008361-35) were randomized into two arms with the same drugs delivered according to different dose intensities. In both arms patients received induction treatment followed by surgery (whenever possible) and/or radiotherapy. The maintenance treatment was given according to the response to the induction treatment (Fig. 1).OS patients enrolled in the ISG/OS2 protocol (EudraCT no. 2011-001659-36) underwent induction chemotherapy with methotrexate 12 g/m^2^ (cycles 1 and 3), and cisplatinum 120 mg/m^2^ and doxorubicin (Adriamycin) 75 mg/m^2^ (cycles 2 and 4). After surgery, patients were stratified to receive a different chemotherapy according to P-glycoprotein (ABCB1) expression and histological response. A total body FDG PET/CT scan was performed in all patients at the time of diagnosis.

### FDG PET/CT imaging

Standardized uptake values (SUVmax) at baseline (SUV1) were calculated for primary tumours and recorded (Fig. 1). SUV1 is reported as median and groups were compared using Student’s *t* test. The threshold for SUV1 was identified as the median plus 1 SD of the baseline. SUV2 was defined as the SUVmax of the primary tumour after induction chemotherapy and is reported as the median (Fig. 1). The threshold for SUV2 was identified as the median plus 1 SD.

### Response assessment

#### Metabolic response

Metabolic responses (percentage reduction in glucose uptake) to primary chemotherapy were calculated according as:[(SUV1 − SUV2)/SUV1)] × 100. Metabolic responses are reported as medians and the threshold was set as the median plus 1 SD.

**Fig. 2:**
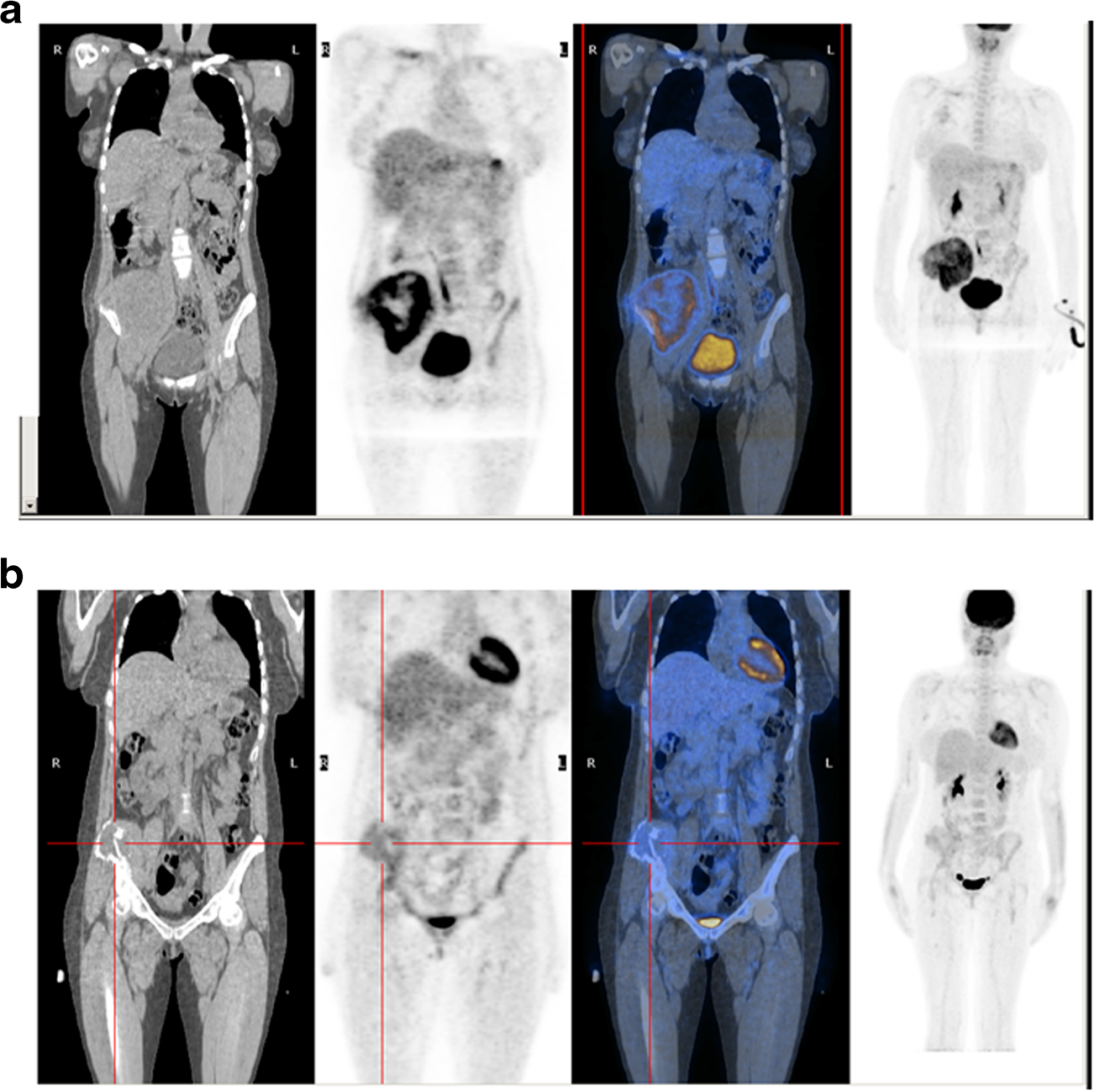
^18^F-FDG PET/CT images in a 37-year-old woman with a left iliac Ewing sarcoma before (**a** SUV1 10) and after (**b** SUV2 3.2) chemotherapy

Each patient received 3.7 MBq/kg of ^18^F-FDG intravenously and the PET/CT scan was performed 60 – 90 min after tracer administration. ^18^F-FDG was produced in our radiopharmacy using a standard technique. PET/CT scans were carried out on a dedicated PET/CT tomograph (Discovery LS; GE Medical System, Waukesha, WI; Fig. 2). PET emission images were collected for 2 min for each bed position from the vertex of the skull to the thighs with inclusion of the upper extremities, and the CT scan was used for nonuniform attenuation correction. CT acquisition parameters were: 120 kV, 80 mA, 0.8 s tube rotation, 3.7 mm slice thickness. To optimize FDG uptake in normal and neoplastic tissues, patients were asked to fast for at least 6 h and were encouraged to void to minimize activity in the bladder before the PET/CT scan. None of the patients had a history of diabetes.

#### Histological/radiological response

Tumour response to chemotherapy was evaluated in all patients. In EWS patients, a histological good response (GR) was defined as grade II/III Picci necrosis in those undergoing surgery [3]. In patients undergoing radiation therapy only, as a local treatment, complete disappearance of the soft tissue component on MRI was considered a GR. In OS patients, the tumour map was analysed histologically in accordance with a previously reported method [27]. The response was considered a GR if tumour necrosis was ≥90 %.

### Laboratory analysis

In all patients a chemistry panel and complete blood count tests were performed before the start of chemotherapy including alkaline phosphatase (ALP) and lactate dehydrogenase (LDH). The normal ranges for ALP at our institution are defined according to gender and age: in males aged <12, 13 – 17 and >17 years the upper limits of the normal ranges are 300, 390 and 129 U/l, and in females are 300, 187 and 104 U/l, respectively.

### Statistical analysis

Event-free survival (EFS) was calculated from the first day of chemotherapy to recurrence (local or distant) or chemotherapy-related death, to the appearance of secondary tumours or to the last follow-up examination. Survival curves were calculated by the Kaplan-Meier method and compared using the log-rank test. In univariate analysis for EFS the following parameters were evaluated: histology (OS vs. EWS), pathological response to chemotherapy (good vs. poor), serum ALP levels (high vs. normal) and LDH levels (high vs. normal), age (adult vs. paediatric) and gender (female vs. male). The relationship between primary tumour SUV1 and SUV2 and tumour histological response/survival was also analysed.

## Results

**Fig. 3:**
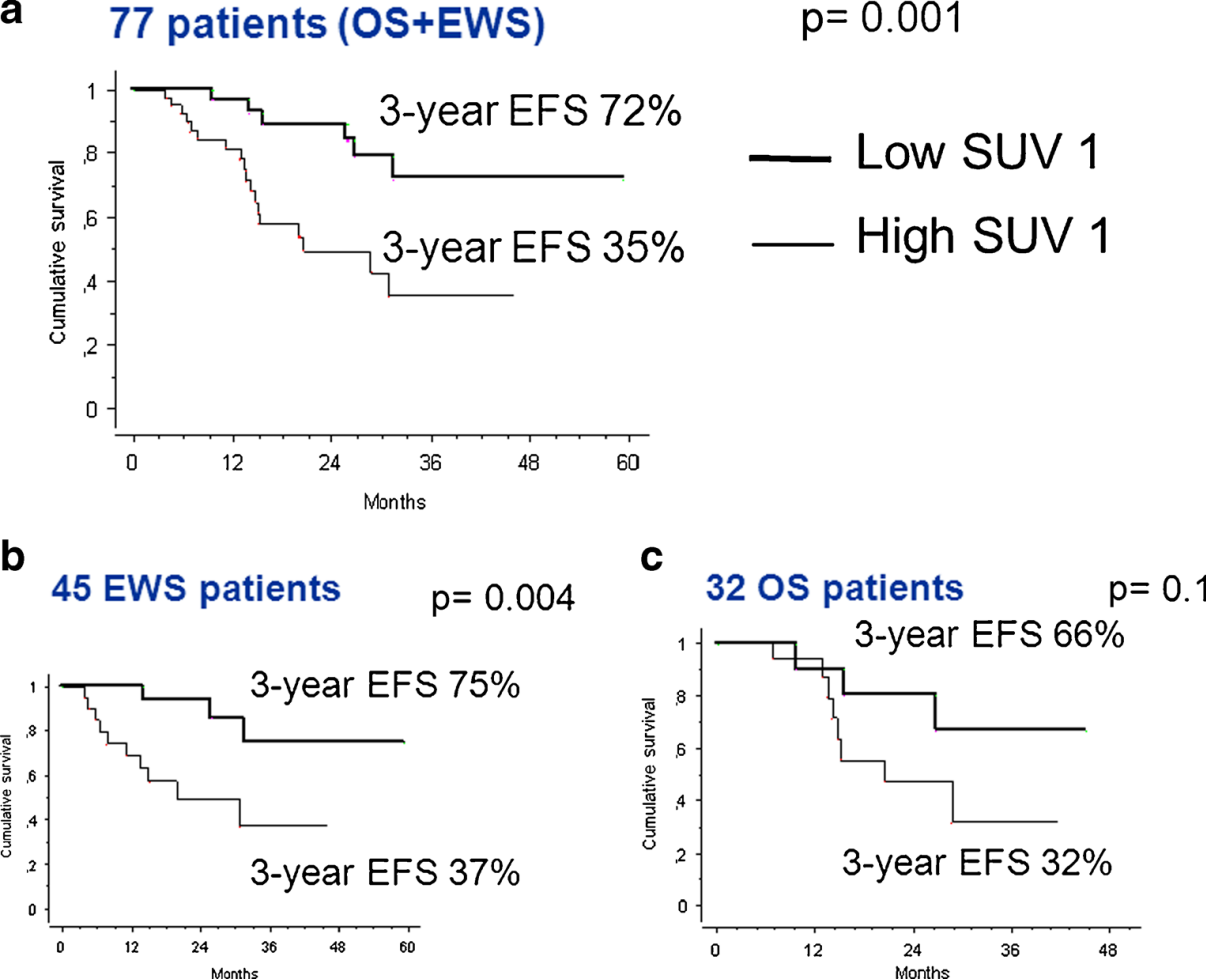
Event-free survival (EFS) at 3 years in relation to SUV1 (low <6, high ≥6) in (**a**) localized bone sarcoma (*OS+EWS*), (**b**) Ewing sarcoma (*EWS*) and (**c**) osteosarcoma (*OS*)

The study included 77 patients, 45 with EWS and 32 with OS. and of these 77 patients, 52 (67 %) were male and 25 (33 %) female (Table 1). The mean age of the patients at presentation was of 17 years (range 3 – 39 years). The primary tumour was located in the extremities in 58 (75 %) of the patients (femur in 26, tibia in 17, fibula in 8, humerus in 5), and in the axial skeleton in 19 patients (pelvis in 14, sacrum in 2, spine in 2, and scapula in 1). The 3-year EFS was 35 % in patients with high SUV1, and 72 % in patients with low SUV1 (*p* = 0.001) overall (Fig. 3a). The median SUV1 was 6.7 (range 0 – 24) overall. The median baseline LDH was 224 U/l (range 61 – 841 U/l), and the median ALP was 109 U/l (10 – 1,006 U/l).

**Table 1:** Clinical characteristics of the patients in the ISG-AIEOP-EW-1 and ISG/OS2 studies

	ISG-AIEOP-EW-1	ISG/OS2
No. of patients	45	32
Age (years)		
Median	16	17
Range	3 – 37	6 – 39
Sex, *n* (%)		
Male	33 (73)	19 (59)
Female	12 (27)	13 (41)
Site, *n* (%)		
Extremities	27 (60)	31 (97)
Pelvis	14 (31)	0 (0)
Spine	4 (9)	0 (0)
Scapula	0 (0)	1 (3)
Alkaline phosphatase, *n* (%)		
Normal	44 (98)	24 (75)
High	1 (2)	8 (25)
Lactate dehydrogenase, *n* (%)		
Normal	33 (73)	24 (75)
High	12 (27)	8 (25)
Local therapy, *n* (%)		
Surgery	28 (62)	32 (100)
External beam radiotherapy	17 (38)	0
SUV1		
Median	5.6	7.8
Range	0 – 17	0 – 24
SUV2		
Median	1.9	2.7
Range	0 – 8	0 – 4.5
Response to induction chemotherapy, *n* (%)		
Good	24 (53)	13 (41)
Poor	21 (47)	19 (59)

**Fig. 4:**
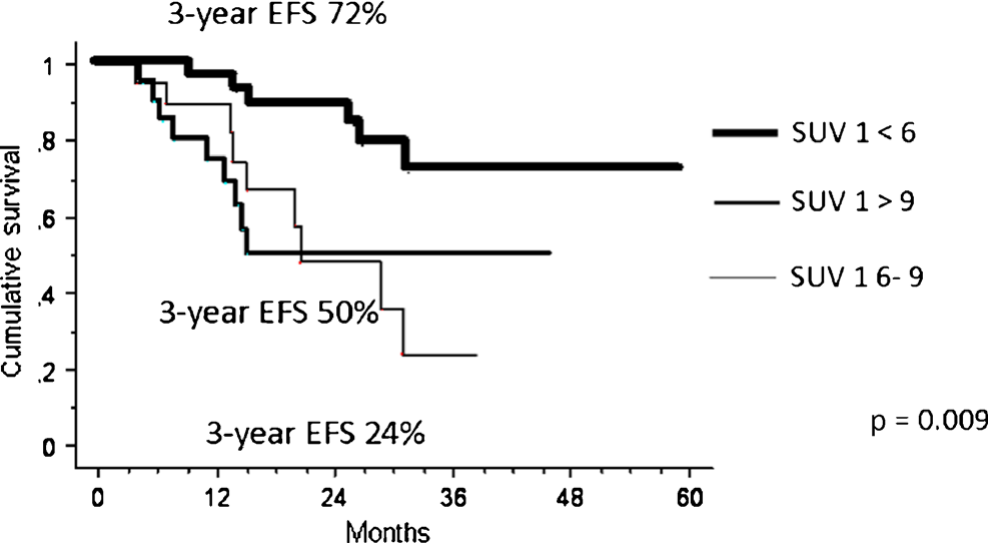
Event-free survival (EFS) at 3 years in relation to SUV1 (<6, 6 – 9, >9) in localized bone sarcoma

The median chemotherapy-induced tumour necrosis was 93 % (range 40 – 100 %). Histological or radiological GR was achieved in 37 of the 77 patients (48 %) overall. The best SUV1 threshold for predicting response was 6 (Fig. 4). With the cut-off set at 6 (SUV1), the GR rate was 29 % in patients with a high SUV1 (≥6) and 69 % in patients with a low SUV1 (<6; *p* = 0.0004) overall.

### Treatment, SUV1, histological response and histological outcome

#### Ewing sarcoma

**Fig. 5:**
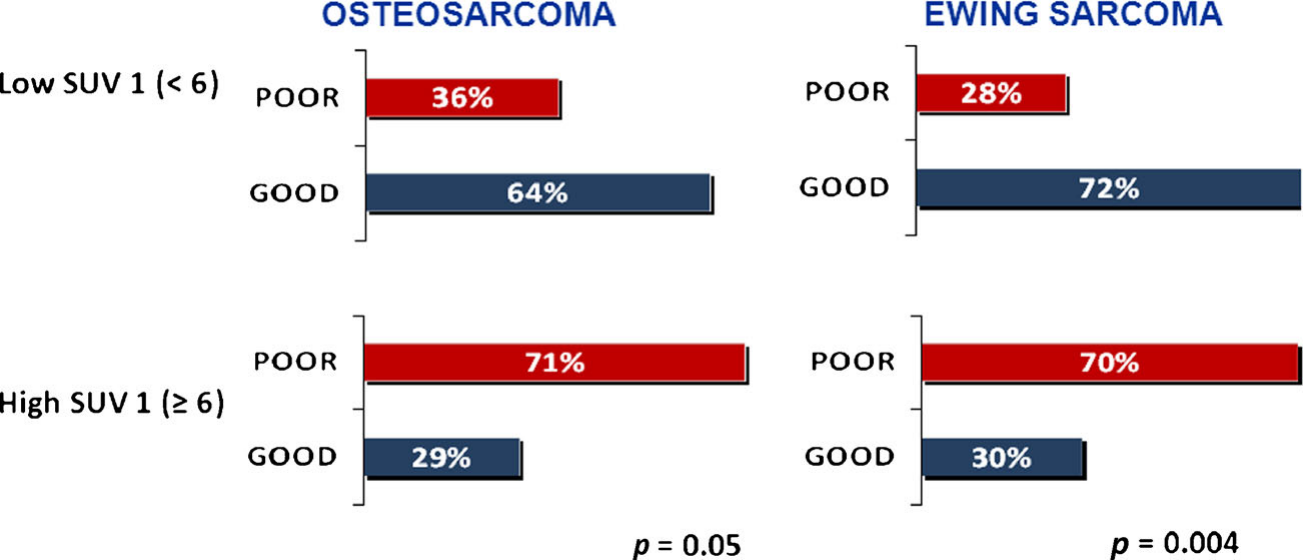
Relationship between primary tumour SUVmax at baseline (SUV1) and tumour histological response

Of the 45 EWS patients 28 (62 %) received surgery and 17 (38 %) received external beam radiotherapy as local treatment. In the latter group, the decision to perform external beam radiotherapy rather than surgery was taken on a case-by-case basis by the orthopaedic surgeons, and was primarily based on age and site. In EWS patients the median SUV1 was 5.6 (range 0 – 17). Histological or radiological GR was achieved in 24/(53 %) of the 45 patients, with a complete histological response in 11 patients (24 %). The GR rate was 30 % in patients with a high SUV1 and 72 % in those with a low SUV1 (*p* = 0.004; Fig. 5). In EWS patients the 3-year EFS was 37 % in those with a high SUV1 and 75 % in those with a low SUV1 (*p* = 0.004; Fig. 3b).

#### Osteosarcoma

All OS patients underwent surgical treatment. Their median SUV1 was 7.85 (range 0 – 24). Histological or radiological GR was achieved in 13 (41 %) of the 32 patients, with a complete histological response in 3 patients (9 %). The GR rate was 20 % in patients with a high SUV1 and 64 % in those with a low SUV1 (*p* = 0.05; Fig. 5). In OS patients the 3-year EFS was 32 % in those with a high SUV1 and 66 % in those with a low SUV1 (*p* = 0.1; Fig. 3c).

### Univariate and multivariate analysis for event-free survival

The results of the univariate analysis for EFS are shown in Table 2. The 3-year EFS was significantly better in patients with a low SUV1 (72 %) than in those with a high SUV1 (36 %, *p* = 0.002), in those with a low ALP level (59 %) than in those with a high ALP level (22 %, *p* = 0.02), and in those with a low LDH level (62 %) than in those with a high LDH level (37 %, *p* = 0.004). However, histology, age and gender were not associated with survival. Combining SUV1 and LDH, four prognostic groups were identified, with worse survival in patients with a high SUV1, independent of LDH level (Table 2).

**Table 2:** Univariate analysis of clinical and pathological variables for event-free survival (EFS) in patients with localized bone sarcoma

Variable		Number of patients	3-year EFS (95 % CI)	*p* value
Overall		77	57 % (41 − 73 %)	
Histology				
Ewing sarcoma		45	57 (38 – 77)	0.5
Osteosarcoma		32	48 (25 – 71)	
Age (years)				
≤18		51	53 (33 – 74)	0.9
>18		26	52 (29 – 76)	
Gender				
Female		37	64 (41 – 86)	0.3
Male		40	46 (26 – 66)	
SUV1				
<6		31	72 (51 – 92)	0.002
≥6		46	36 (14 – 57)	
LDH (baseline)				
Low		55	62 (44 – 80)	0.004
High		22	37 (13 – 61)	
ALP (baseline)				
Low		68	59 (43 – 75)	0.02
High		9	22 (0 – 57)	
SUV1/LDH (baseline)				
<6	LDH low	29	78 (59 – 98)	0.00007
	LDH high	6	56 (7 – 100)	
≥6	LDH low	36	36 (6 – 67)	
	LDH high	16	34 (10 – 50)	

In the multivariate analysis, SUV1 was the only independent pretreatment prognostic factor to retain statistical significance (*p* = 0.017; Table 3).

**Table 3:** Multivariate analysis of clinical and pathological variables for event-free survival in patients with localized bone sarcoma

Variable	Relative risk	95 % CI	*p* value
Histology			
Osteosarcoma	1		0.623
Ewing sarcoma	1.25	0.50 – 3.11	
SUV1			
Low	1		0.017
High	3.25	1.24 – 8.52	
LDH			
Low	1		0.07
High	2.18	0.93 – 5.14	
ALP			
Low	1		0.20
High	2.06	0.68 – 6.23	

### Metabolic response: SUV2

The results discussed in this section relate exclusively to the 25 patients with localized EWS and 12 patients with localized OS who underwent a PET scan for reassessment after induction chemotherapy.

#### Ewing sarcoma

**Fig. 6:**
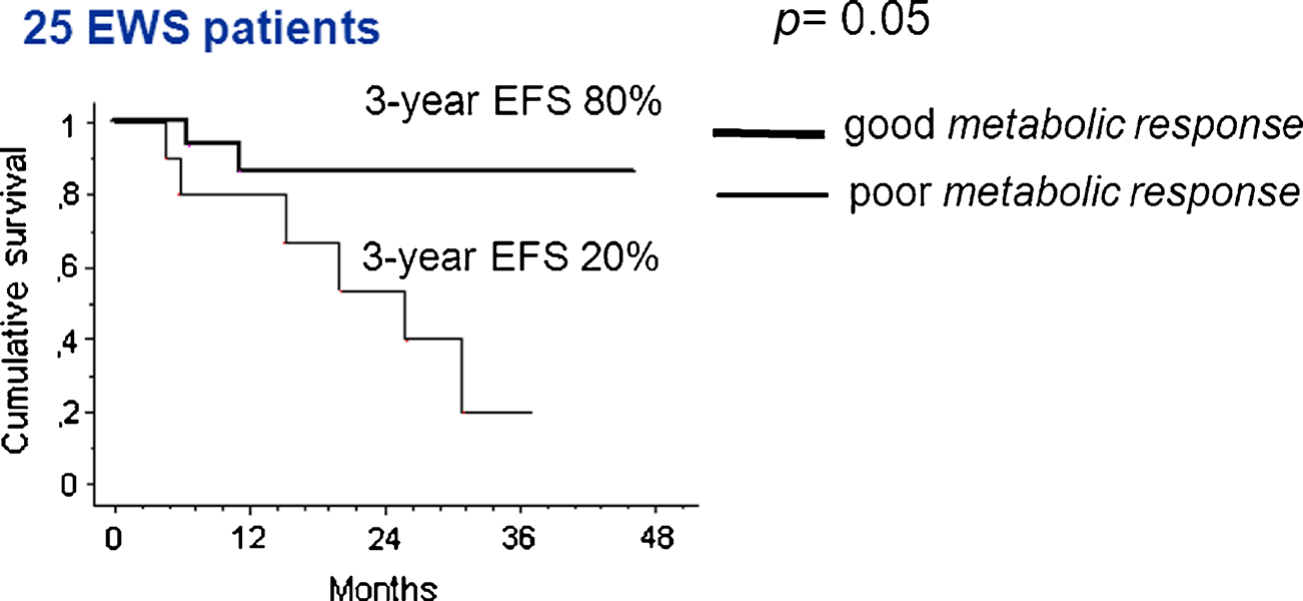
Event-free survival (EFS) at 3 years according to metabolic response after induction chemotherapy in patients with localized Ewing sarcoma

The median SUV2 was 1.9 (range 1 – 8), with a median metabolic response of 59 % (range 7 – 99 %). The GR rate was 20 % in patients with a high SUV2 (≥3) and 67 % in those with a low SUV2 (<3; *p* = 0.02). The 3-year EFS was 80 % in patients with a good metabolic response (reduction in SUV of ≥55 %) and 20 % in those with a poor metabolic response (reduction in SUV of <55 %; *p* = 0.05; Fig. 6).

#### Osteosarcoma

The median SUV2 was 2.7 (range 0 – 4.5) with a median metabolic response of 48 % (range 4 – 99 %). The GR rate was 50 % in patients with a high SUV2 (≥3) and 75 % in those with a low SUV2 (<3; *p* = 0.4). The 3-year EFS was 20 % in patients with a good metabolic response (reduction in SUV of ≥55 %) and 100 % in those with a poor metabolic response (reduction in SUV of <55 %; *p* not assessable).

## Discussion

A histological response to neoadjuvant chemotherapy is one of the strongest prognostic factors in patients with OS [27–29] and EWS [3, 30]. Histological response has significant limitations, including the ability to assess it only after 10 to 15 weeks of initial chemotherapy. The current study represents the largest series of patients with bone sarcomas in which the prognostic value of pretreatment SUV1 was assessed (Table 4). This study showed an association between SUV1 and histological/radiological response in patients with OS and in those with EWS. SUV1 was also predictive of outcome in terms of EFS in patients with EWS, with a trend in patients with OS. This is in contrast with Hawkins et al. paper, founding no differences in EFS at 4-year (63 % vs. 73 %, p 0.4), in a series of 34 localized OS, with a median age of 15 [31].

**Table 4:** Studies on the use of FDG PET in localised bone sarcoma including outcome correlation and SUV thresholds

Reference	Year	Median age (years)	No. of patients	Histology	SUV1 (median)	End-point	SUV threshold	Survival (%)	*p* value
[7]	2005	18.7	36^a^	Ewing sarcoma	7.9	4-year PFS	<6	62	0.47
							≥6	52	
[31]	2009	15	40^b^	Osteosarcoma	6.8	4-year PFS	>6	73	0.41
							≤6	63	
[15]	2002	13.3	18	Osteosarcoma	8.2	ND			
			15	Ewing sarcoma	5.3	ND			
[22]	2009	14	70	Osteosarcoma	8	ND			
[20]	2013	21	26	Osteosarcoma	9.2	ND			
[16]	2002	14	26	Osteosarcoma	12.6	3-year EFS	12.6	90 28	<0.005
[25]	1996	19	4	Osteosarcoma	5.8	ND			
		16	1	Ewing sarcoma	5.8				
[19]	2006	17.5	10	Osteosarcoma	9.1	ND			
[32]	2016	12.6	50	Ewing sarcoma	5	ND			
Palmerini et al.	this study	17	32	Osteosarcoma	7.8	3-year EFS	<6	66	0.1
							≥6	32	
		16	45	Ewing sarcoma	5.6	3-year EFS	<6	75	0.004
							≥6	37	

*EFS* Event-free survival, *PFS* Progression-free survival, *ND* not done

^a^12 metastatic

^b^6 metastatic

To our knowledge, this is the first report of an association between SUV1 and EFS in patients with EWS. Multivariate analysis confirmed SUV1 as and independent prognostic factor, and SUV1 should be take into consideration together with other well-known prognostic factors such as patient age, and tumour site and size [3, 29]. The strength of this study was the homogeneity of treatment, including the duration of neoadjuvant chemotherapy, the surgical team and pathological assessment, all important determinants of EFS that could be confounding factors in assessing the predictive value of FDG PET. The concordance between a good histological response (>90 % tumour necrosis in OS patients and Picci II/III necrosis in EWS patients) and SUV1 (<6) in this study was very robust, confirming the findings in smaller series [15, 16, 32, 33], and is in contrast with the findings of a study performed in America [31]. Differences in patient characteristics (age), treatment and histological examination assessment could explain these conflicting results.

FDG PET may have several potential clinical uses. First, treating physicians may be able to identify patients who are more likely to have a less favourable histological response to neoadjuvant chemotherapy. Such patients would be candidates for more aggressive front-line chemotherapy. Nonrandomized clinical trials have suggested that augmentation of chemotherapy in response to a poor histological response can improve outcome [34, 35]. However, this observation has been refuted in other studies [36, 37], including the randomized European and American Osteosarcoma Study Group trial (EURAMOS-1), in which the hypothesis that a response-adapted therapy would provide a survival benefit was not confirmed [38].

It is possible that SUV1-adapted chemotherapy would be more useful than histological response-adapted chemotherapy, because of the ability to identify chemotherapy-resistant patients from the beginning of therapy. Furthermore, combining SUV1 level with one or more already validated prognostic factors such as patients age or LDH level could eventually identify a ‘prognostic score’, similar to those used in other malignancies such as breast cancer. SUV2 seems exclusively useful in EWS patients. In our study SUV2 was able to predict histological response, as shown in a paediatric series [32], and might be used for example by surgeons to plan a local treatment approach. Furthermore, a good metabolic response was associated with best survival in EWS patients (80 % 3-year EFS in patients with a reduction in SUV2 of ≥55 %, in contrast to 20 % in patients with a poor metabolic response). In OS patients metabolic response did not appear to be a valid prognostic tool, in contrast by previously reported findings in children and young adults [31]. The major limitation of this subanalysis was the small sample size.

**Fig. 7:**
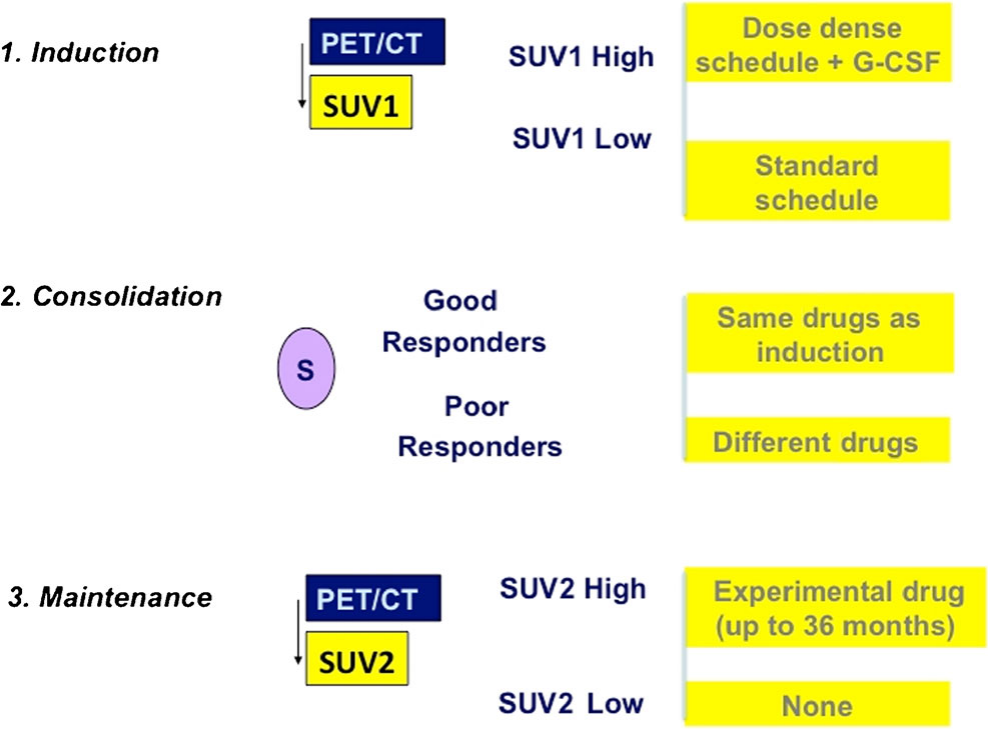
Treatment algorithm for the use of FDG PET/CT in patients with localized Ewing sarcoma (*G-CSF* granulocyte colony-stimulating factor)

In conclusion, this is an important study on the role of PET in the management of localized bone sarcomas. The study demonstrated that an SUV1 <6 before neoadjuvant chemotherapy was an independent prognostic factor for EFS. The study confirmed that FDG PET imaging can complement histological response. Only in EWS patients was a good metabolic response associated with the best survival, and a treatment algorithm based on FDG PET/CT data could be proposed in patients with localized EWS (Fig. 7). Additional research should prospectively address the impact on survival of treatment modification in patients who are at greater risk of disease recurrence.
